# A Trimethoprim-Based Chemical Tag for Live Cell Two-Photon Imaging

**DOI:** 10.1002/cbic.200900731

**Published:** 2010-03-09

**Authors:** Sarah S Gallagher, Chaoran Jing, Darcy S Peterka, Mariam Konate, Richard Wombacher, Laura J Kaufman, Rafael Yuste, Virginia W Cornish

**Affiliations:** [a] Department of Chemistry, Columbia University3000 Broadway, MC 3111, New York, NY 10027 (USA) Fax: (+1) 212-932-1289 E-mail: vc114@columbia.edu; [b] Howard Hughes Medical Institute, Department of Biological Sciences, Columbia University1212 Amsterdam Avenue, Box 2435, New York, NY 10027 (USA)

**Keywords:** chemical tags, fluorescence, live cell imaging, microscopy, proteins

Two-photon excitation results from the near simultaneous absorption of two relatively low-energy photons by a fluorophore, causing a transition to an excited state with an energy difference close to that of the combined two photons (Figure 1 A).^1^ For most biologically relevant two-photon fluorophores, the excitation light used is in the near infrared (NIR) region.^2^ Several characteristics of two-photon microscopy make it an attractive technique for biological imaging. Two-photon absorption is strongly dependent on the intensity of the incident light, and therefore, excitation only takes place in a small volume centered at the focal plane, giving inherent sectioning and greatly reducing background fluorescence from out-of-focus excitation. Because the two-photon excitation is limited to the focal plane, photodamage and bleaching to the sample are minimized.^3^ Additionally, the greatly reduced absorption and scattering of NIR light allows for deeper penetration into biological samples.^4^ Here we report the development of a trimethoprim (TMP) chemical tag suitable for two-photon imaging in live cells; this adds a robust two-photon fluorophore to the repertoire of labels available with this technology.

**Figure 1:**
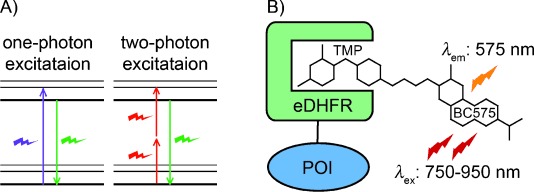
A trimethoprim (TMP)-based chemical tag for two-photon imaging. A) Energy level diagram illustrating one- and two-photon excitation. Two-photon excitation occurs from the near simultaneous absorption of two photons that are approximately half of the energy of the corresponding one-photon absorption. B) Cartoon depicting the TMP-eDHFR labeling system. An intracellular protein of interest (POI) is fused to a receptor domain, eDHFR, that specifically binds the TMP-two-photon fluorophore (BC575) conjugate.

With the chemical tags, rather than tagging the protein of interest with a fluorescent protein (FP), the protein of interest is tagged with a polypeptide that is subsequently modified with an organic fluorophore.^5^–^8^ Thus, the chemical tags combine the selectivity of genetic encoding with a modular organic fluorophore.^9^–^11^ We have previously established that proteins tagged with *E. coli* dihydrofolate reductase (eDHFR) can be labeled noncovalently^12^–^14^ (and recently covalently)^15^ with cell-permeable trimethoprim (TMP)–fluorophore conjugates in mammalian cells. The TMP-based tag is an attractive chemical tag because eDHFR is small in size (18 kD), TMP analogues are straightforward to synthesize, and labels based on the TMP antibiotic have no apparent cross reactivity or toxicity in mammalian cells.^16^, ^17^ Currently, the TMP-tag is one of the few available chemical tags capable of specifically labeling intracellular proteins in living cells.^14^, ^18^ To our knowledge, while a variety of organic fluorophores, quantum dots,^19^ and lanthanide chelates^17^ have been adapted for the chemical tag technology, currently there are no reported chemical tags optimized for two-photon imaging.

Key to the design of a TMP-tag for two-photon microscopy was selection of a two-photon fluorophore for conjugation to TMP that would have the desired photophysical properties yet also be cell permeable. The fluorophore must have a high two-photon action (TPA) cross section, which is the product of the two-photon absorption cross section and the fluorescence quantum yield. Second, when linked to TMP, the two-photon fluorophore must behave well in the cell; it must be both cell-permeable and not partition into lipid vesicles.^14^ In this study, we employed a 2*H*-benzo[*h*]chromene-2-one derivative, which we refer to as BC575. This fluorophore was recently reported by Kim and co-workers to have a high TPA cross-section (67 Goeppert–Mayer, or GM [1 GM=10^−50^ cm^4^ s per photon]), and to be cell permeable.^20^, ^21^

The retrosynthetic analysis of the TMP-BC575 conjugate is shown in Scheme 1. As previously reported,^14^ the synthesis began with the selective hydrolysis of the 4′-methoxy group of TMP in 48 % HBr; this resulted in a phenol, which was alkylated with ethyl 5-bromovalerate. Saponification of the ester produced a carboxylic acid, which was coupled to mono-Boc protected 1,13-diamino-4,7,10-trioxatridecane and then deprotected to generate a free amine. The synthesis of BC575 was completed in four steps.^20^ The amino group of 6-aminotetralone was methylated by iodomethane to yield the corresponding dimethylaniline, which was formylated by treatment with ethyl formate. The ring was aromatized and condensed with dimethyl malonate, giving BC575. The methyl ester was then hydrolyzed, and the resulting carboxylic acid was coupled to the free amine of the TMP portion by using standard peptide-coupling conditions. Thus, the TMP-BC575 conjugate was synthesized from two components in six linear steps in 0.3 % overall yield from 6-amino-3,4-dihydronapthalen-1(2 *H*)-one, the longest linear route.

**Scheme 1:**
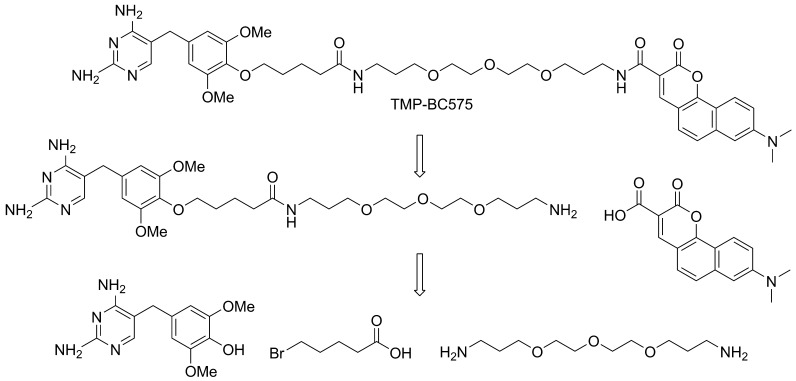
>Retrosynthetic analysis of the TMP-BC575 conjugate.

To verify the performance of BC575, its two-photon excitation and emission spectra were first measured in vitro. Rectangular glass capillaries (300×50 microns) were filled with a 100 μm solution of BC575 dissolved in DMF. The capillaries were sealed and fixed on a coverslip immersed in a drop of water. The two-photon fluorescence data were acquired by using a custom-made two-photon laser scanning microscope based on the Olympus FV-300 system (FV-300 side-mounted to a BX50WI microscope with a 60×, 1.1 numerical aperture, water immersion objective) and a Ti:sapphire laser (Chameleon Ultra II, Coherent).^22^ Fluorescence of BC575 was evaluated following two-photon excitation by using wavelengths from 750–1050 nm, which are typically used for two-photon biological imaging applications. Additionally, we measured the fluorescence intensity of a 100 μm sample of rhodamine B in H_2_O by using the same microscope setting; this allowed for the direct comparison of BC575 to rhodamine B, which is known to be a bright two-photon dye.^23^ The total signal intensities were then determined by using the program ImageJ^24^ and were used to calculate the normalized fluorescence intensities for the dyes (Figure 2 A).^23^, ^25^ The results show that BC575 has appreciable two-photon excited fluorescence from 750–950 nm and confirms the utility of BC575 for two-photon microscopy.

**Figure 2:**
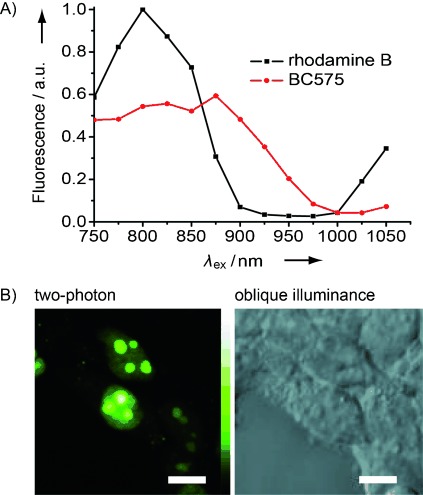
Characterization of the two-photon fluorescent chemical tag TMP-BC575. A) In vitro characterization of two-photon excitation of BC575. To evaluate the utility of the fluorophore for two-photon imaging, the two-photon excited fluorescence intensity (in arbitrary units) was measured for a 100 μm solution of BC575 (red) from 750–1050 nm, the total fluorescent emission between 490 nm and 630 nm was collected by using a Chroma bandpass filter. For comparison, the spectrum for rhodamine B (black) was measured by using the same microscope setting. The fluorescence intensity of rhodamine B and BC575 were both normalized to the fluorescence signal maximum of rhodamine B at 800 nm. B) Live-cell two-photon imaging of intracellular proteins using the TMP-BC575 conjugate, scale bar: 10 μm. HEK293 cells transfected with nucleus-targeted eDHFR were incubated with 1 μm TMP-BC575 for 10 min, washed in HEPES buffer, and then imaged using two-photon microscopy. Two-photon micrograph following excitation at 940 nm (left) and single-photon oblique illumination image (right) are presented. Transfected cells incubated with TMP-BC575 show clear nuclear staining.

Having demonstrated in vitro that BC575 is a good two-photon fluorophore, we then evaluated the utility of the TMP-BC575 conjugate for in vivo imaging. Human embryonic kidney (HEK) 293 cells were seeded on coverslips and transiently transfected with vector DNA encoding nucleus-targeted eDHFR.^13^ The cells were then incubated in media containing 1 μm TMP-BC575 for 10 min at 37 °C, washed and then imaged by oblique illumination and by two-photon microscopy by using excitation at 940 nm (Figure 2 B). Notably, the transfected cells showed distinct nuclear labeling without any significant staining of the cytoplasm or untransfected cells. Comparison of the fluorescence signal intensities from two-photon excitation of both transfected and untransfected cells incubated with TMP-BC575 verified that the conjugate can be used to label proteins of interest with high signal-to-noise (Figure S5 in the Supporting Information). These results establish that, significantly, the TMP-BC575 conjugate has the combination of cell permeability and two-photon brightness necessary for two-photon live cell imaging.

Thus, TMP-BC575 is an immediately viable tool for imaging proteins in live cells by using two-photon microscopy. This two-photon fluorophore expands the TMP-tag tool kit, adding to the value of this modular-labeling technology. A protein of interest can be tagged with eDHFR, and different labels can then be swapped in, allowing the protein to be readily analyzed by multiple techniques. While cell permeability and lipid partitioning appear to be tag dependent, these experiments suggest BC575 might also be compatible with other chemical tags. Given its broad excitation maxima and distinct emission wavelength, TMP-BC575 offers an alternative to other FPs for multicolor two-photon imaging with enhanced green fluorescent protein (EGFP; see Figures S2 and S3 in the Supporting Information),^26^–^28^ Alternatively, an orthogonal chemical tag with a nonoverlapping two-photon fluorophore could be developed for multicolor two-photon microscopy. Furthermore, TMP-BC575 might be advantageous in applications in which the larger FP fusion interferes with biological function or where the reversibility of the fluorophore labeling can be exploited. The next step is to challenge TMP-BC575 to label a variety of intracellular proteins. We are also exploring the utility of TMP-BC575 for two-photon imaging of tissue sections and live animals. This work opens the door for two-photon imaging with chemical tags and further illustrates how the modular organic label of chemical tags can be harnessed for state-of-the-art biological imaging.

## Experimental Section

The complete details of TMP-BC575 synthesis and characterization of its photophysical properties, cell culture condition and staining methods, as well as experimental details of two-photon microscopy are reported in the Supporting Information.

## Supplementary material

Detailed facts of importance to specialist readers are published as ”Supporting Information”. Such documents are peer-reviewed, but not copy-edited or typeset. They are made available as submitted by the authors.
