# Supplementary material for: A Trimethoprim-Based Chemical Tag for Live Cell Two-Photon Imaging
Source: Chembiochem. 2010 Mar 9;11(6):782–4. doi: 10.1002/cbic.200900731 (PMC2954500; doi:10.1002/cbic.200900731)
Supplement: Supplementary file 1 [file cbic0011-0782-SD1.pdf]

CHEMBIOCHEM

Supporting Information

© Copyright Wiley-VCH Verlag GmbH & Co. KGaA, 69451 Weinheim, 2010

A Trimethoprim-Based Chemical Tag for Live Cell Two-Photon Imaging

Sarah S. Gallagher,[a] Chaoran Jing,[a] Darcy S. Peterka,[b] Mariam Konate,[a] Richard Wombacher,[a] Laura J. Kaufman,[a] Rafael Yuste,[b] and Virginia W. Cornish*[a]

cbic_200900731_sm_miscellaneous_information.pdf

Chemical synthesis

General methods. Unless otherwise noted, reagents were obtained from Aldrich and used without further purification. All moisture- or oxygen-sensitive reactions were performed under argon in flame- or oven-dried glassware. Organic extracts were dried over magnesium sulfate or sodium sulfate. Analytical thin layer chromatography (TLC) was performed on silica gel (Whatman LHPKF Silica Gel 60Å) and visualized by UV light (254 nm). All column chromatography was flash chromatography carried out on silica gel (EM Science Silica Gel 60Å), and all eluents used are reported in volume:volume ratios. Nuclear Magnetic Resonance (NMR) spectra were recorded on a Bruker 400 (400 MHz) Fourier Transform (FT) NMR spectrometer at the Columbia University Department of Chemistry NMR facility. Spectra were taken in deuterated solvents as indicated, using the solvent residual peaks as reference. ^{1}H-NMR resonances are reported in units of parts per million (ppm) downfield from trimethylsilane and are tabulated in the following order: multiplicity (s, singlet; d, doublet; t, triplet; m, multiplet; br, broad), number of protons. Mass spectra (MS) were recorded at the Columbia University Department of Chemistry Mass Spectral laboratory. Fast Atom Bombardment (FAB) high resolution mass spectra (HRMS) were recorded on a JMS-HX110A mass spectrometer.

Synthesis design. The synthesis of the TMP-BC575 molecule is shown in Scheme S1.

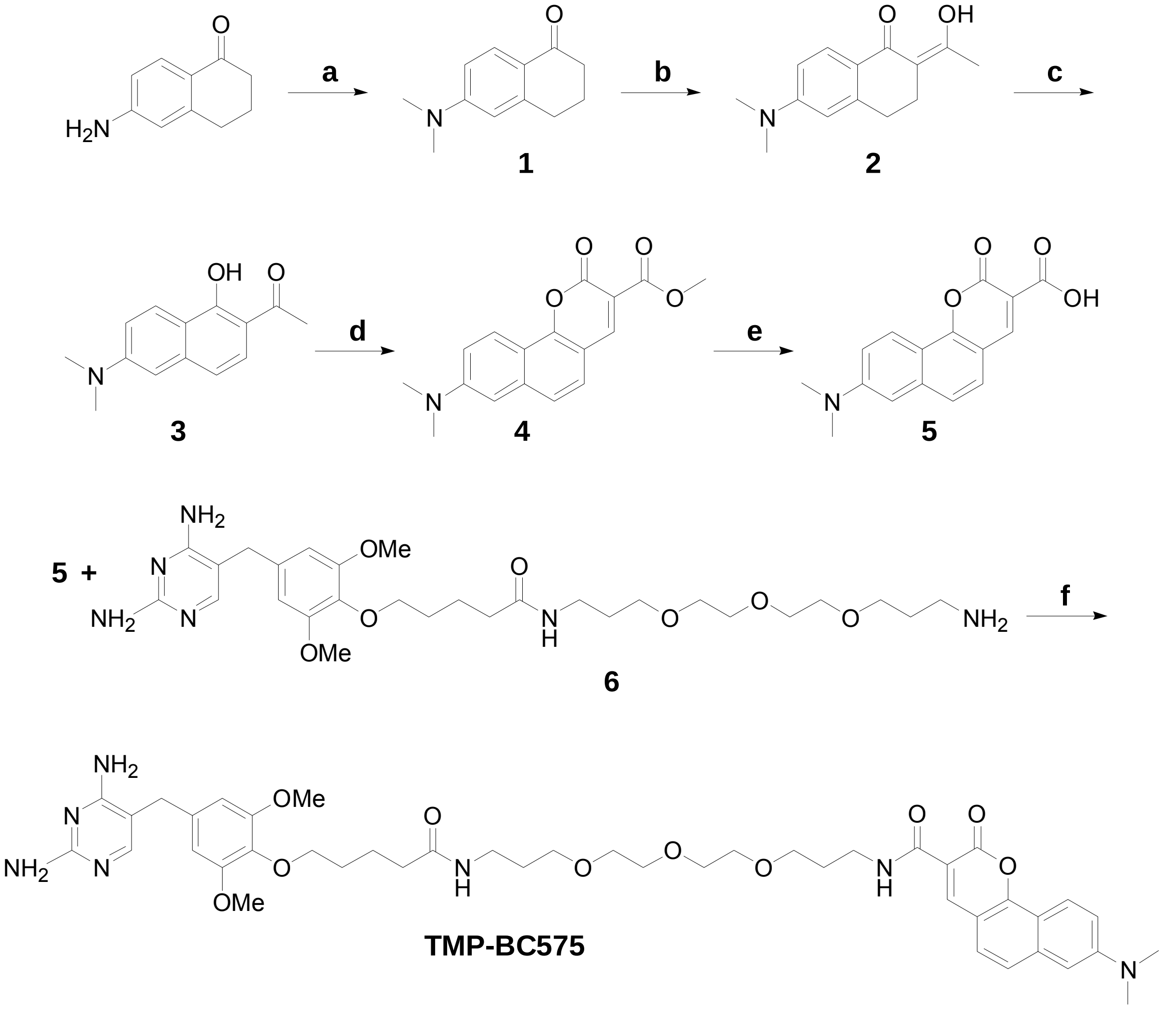

Scheme S1: Synthesis of TMP-BC575. Reaction conditions: a. K_2CO_3, MeI, CH_3CN, 50°C, 16 h, 21%; b. ethyl formate, NaOMe, benzene, 62%; c. DDQ, 1,4-dioxane, 69%; d. dimethyl malonate, piperidinium acetate, reflux, toluene, 16 h, 16%; e. i. KOH, EtOH, 5 h, ii. HCl, 66%; f. PyBOP, DIEA, DMF, 31%.

*Synthesis of **1**.* 6-Amino-3,4-dihydronapthalen-1(2H)-one (0.34 g, 2.1 mmol) and K_2CO_3 (1.0 g, 7.4 mmol) were added to a flame-dried 50 mL round bottom flask and then suspended in anhydrous CH_3CN (11 mL). Iodomethane (4.1 g, 29 mmol) was added and the reaction mixture was stirred under argon at 50 ˚C for 16 hr in the dark. Then CH_2Cl_2 (10 mL) and H_2O (10 mL) were added and the mixture was stirred. The aqueous layer was extracted with CH_2Cl_2 (2 x 10 mL) and the organic layers were combined, dried with $MgSO_4$, filtered, and concentrated to dryness. The product was then purified by silica gel flash chromatography (8:2 Hex:EtOAc + 1% MeOH) to give 76 mg (21% yield) of compound **1**: R_f = 0.32 in 9:1 CH_2Cl_2:MeOH. ^1H NMR (400 MHz, $CDCl_3$) δ ppm: 7.86 (d, 1H), 7.73, (d, 1H), 6.60 (dd, 1H), 6.40 (d, 1H), 3.05 (s, 6H), 2.81 (t, 2H), 2.50 (t, 2H).

Synthesis of **2.** Sodium methoxide (1.2 g, 22 mmol), ethyl formate (0.94 mL, 12 mmol), and anhydrous benzene (9 mL) were added to a flame-dried round bottom flask. Then, compound **1** (0.95 g, 5.0 mmol) was dissolved in anhydrous benzene (6 mL) and was added dropwise to the reaction mixture with stirring. The reaction mixture was stirred overnight under argon at RT in the dark. After 16 hr, ether (20 mL) and H_2O (20 mL) were added to the reaction mixture and the layers were separated. The aqueous layer was acidified to pH 4 with hydrobromic acid. The aqueous layer was then extracted with ether (20 mL). The organic layer was washed with H_2O (20 mL) and brine (20 mL) and then dried over $MgSO_4$, filtered, and concentrated to dryness to yield 0.68 g (62%) of compound **2**: R_f = 0.52 in DCM; ^{1}H NMR (400 MHz, $CDCl_3$) δ ppm: 7.94 (d, 1H), 6.60 (dd, 1H), 6.41 (d, 1H), 3.04 (s, 6H), 2.86 (t, 2H), 2.55 (t, 2H), 2.07 (m 2H); MS(FAB^+), m/z 217.38 (M^+), calculated 217.11.

Synthesis of **3.** To a flame-dried, 25 mL round bottom flask, compound **2** (38 mg, 0.17 mmol), DDQ (43 mg, 0.19 mmol), and anhydrous dioxane (5 mL) were added. The reaction was stirred at RT under argon in the dark for 20 hr. The hydroquinone was removed by filtration and the solvent of the filtrate was removed *in vacuo.* The product was purified by column chromatography on silica gel (9:1 Hex:EtOAc) to yield compound **3** (26 mg, 69%): R_f = 0.4 in 20:1 Hexane:EtOAc; ^{1}H NMR (300 MHz, $CDCl_3$) δ ppm: 12.82 (s, 1H), 9.76 (s, 1H), 8.24 (d, 1H), 7.29 (d, 1H), 7.10-7.04 (m, 2H), 6.74 (d, 1H), 3.12 (s, 6H); MS(FAB^+), m/z 215.36 (M^+), calculated 215.09.

Synthesis of **4.** A mixture of compound **3** (26 mg, 0.12 mmol), dimethyl malonate (14 µL, 0.12 mol), and a catalytic amount of piperidinium acetate in toluene (4.3 mL) was refluxed in a Dean-Stark trap for 16 hr. The mixture was cooled and the solvent was removed *in vacuo*. The product was purified by column chromatography on silica (6:1 Hex:EtOAc) to yield compound **4** (5.9 mg, 16%): R_f = 0.3 in 5:2 hexane:EtOAc; ^{1}H NMR (300 MHz, $CDCl_3$) δ ppm: 8.59 (s, 1H), 8.37, (d, 1H), 7.35 (dd, 2H), 7.14 (dd, 1H), 6.80 (d, 1H), 3.94 (s, 3H), 3.13 (s, 6H); MS(FAB^+), m/z 297.35 (M^+), calculated 297.10.

Synthesis of **5**. Compound **4** (15 mg, 51 µmol) and KOH (5.8 mg, 100 µmol) were suspended in EtOH (4.0 mL). The reaction mixture was stirred at RT in the dark for 5 hr. The reaction mixture was then diluted with ice water and the pH was adjusted to 3 using concentrated HCl and a dark red precipitate formed. The crystals were collected by filtration giving compound **5** (9.6 mg, 66%).

Synthesis of **6**. We have previously described the synthesis of compound **6**.[1]

Synthesis of TMP-BC575. Compound **6** (5.3 mg, 9.0 µmol), compound **5** (3.2 mg, 11 µmol), and PyBOP (7.4 mg, 14 µmol) were added to a flame-dried vial and dissolved in DMF (0.4 mL). DIEA (7.6 µL, 44 µmol) was added to the vial and the reaction was stirred under nitrogen overnight at RT in the dark. Then, the solvent was removed *in vacuo* and the product was purified by column chromatography on silica (12:1

CH_2Cl_2:MeOH). The product was then purified by HPLC (15:85 H_2O with 0.1% TFA : CH_3CN to H_2O with 0.1% TFA) to yield TMP-BC575 (2.3 mg, 31 %): R_f = 0.16 in 20:1 DCM: MeOH; ^1H NMR (400 MHz, CD_3OD) δ ppm: 8.79 (s, 1H), 8.26 (d, 1H), 7.51 (dd, 2H), 7.31 (dd, 1H), 7.20 (s, 1H), 6.97 (d, 1H), 6.52 (s, 2H), 3.89 (t, 2H), 3.77 (s, 6H), 3.70-3.48 (m, 16H), 3.24 (t 2H), 3.15 (s, 6H), 2.24 (t, 2H), 1.89 (t, 2H), 1.80-1.67 (m, 6H); MS(FAB^+), m/z 844.58 (MH^+); HRMS(FAB^+), m/z 844.4269 (MH^+), calculated 844.42. The ^1H-NMR spectrum the TMP-BC575 conjugate is shown in Figure S1.

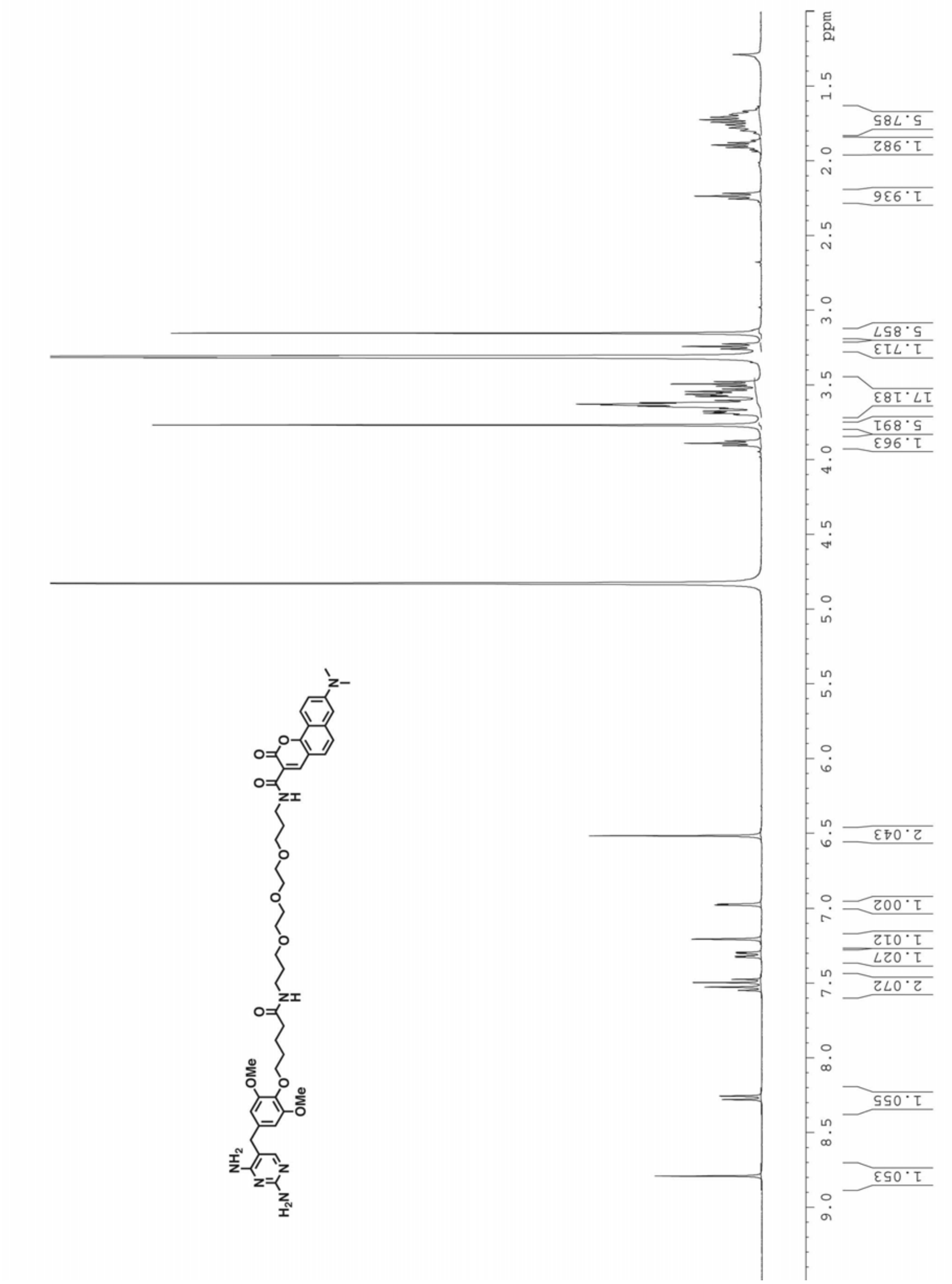

Figure S1. ^{1}H NMR spectrum of the TMP-BC575 conjugate (400MHz, CD_3OD).

Spectrospic measurements

UV-visible absorption spectra. One-photon absorption spectra were recorded on a Spectramax Plus384 UV-visible spectrophotometer (Molecular Devices). The absorption spectra of BC575 and TMP in DMF solution are shown in Figure S2 A).

One-photon excited fluorescence spectrum. One-photon fluorescence spectrum (normalized fluorescence intensity in arbitrary units) of BC575 was recorded on a LS 55 luminescence spectrometer (Perkin Elmer) from excitation at 453 nm. The fluorescence spectrum of 0.25 μM BC575 in water is shown in Figure S2 B).

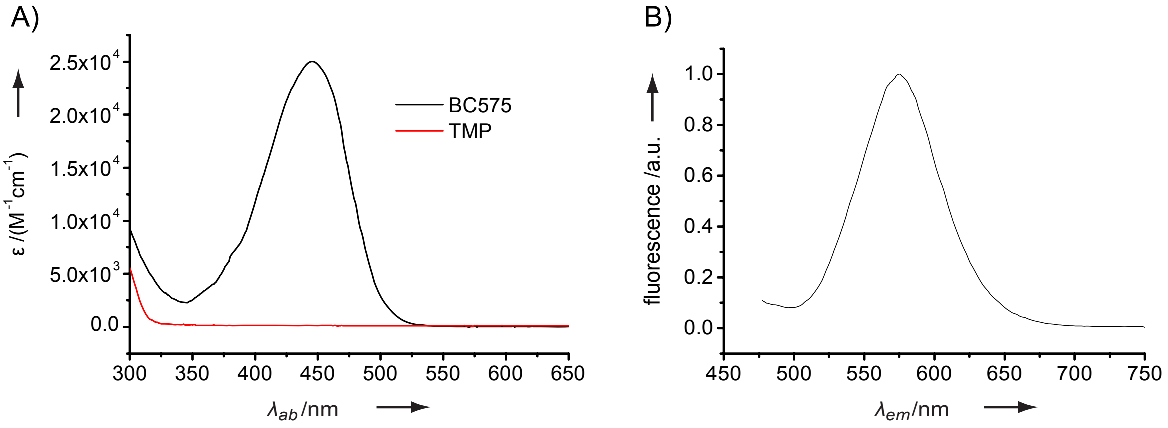

Figure S2. *In Vitro* Characterization of the One-Photon Absorption Spectra of BC575. A) Determination of the molar extinction coefficient of BC575 and TMP in DMF. B) Normalized fluorescence spectrum of BC575 in H_2O.

Two-photon absorption spectra. The two-photon fluorescence data was acquired using a custom-made two-photon laser scanning microscope based on the Olympus FV-300 system (FV-300 side-mounted to a BX50WI microscope with a 60×, 1.1NA, water immersion objective) and a Ti:sapphire laser (Chameleon Ultra II, Coherent).[2] Fluorescence was detected with a top-mounted Hamamatsu H7422-P40 PMT connected to a Stanford Research System SR570 preamplifier whose output was connected to the Fluoview system, and simultaneously recorded with a DAQ card (National Instruments PCI-6259). Rectangular glass capillaries (inner dimensions 300 x 50 microns) filled with 100 μM of BC575 in DMF and 100 μM of Rhodamine B in water, respectively, were sealed and fixed on a cover slide and covered by a drop of water. The objective was immersed in the drop of water and a series of fluorescence images were collected for excitation from 750 to 1050 nm (Delta nm = 25). The total fluorescent emission between 490 nm and 630 nm (Chroma bandpass filter 560/140) was collected and the total signal intensities were quantified using ImageJ, and were normalized to changes in laser power and laser pulse width, using equation (1).[3] The fluorescence intensity before and after adjustment are denoted as F_{mea} and F_{adj}, respectively. P is the laser power on the sample; τ is laser pulse width

on sample; λ is excitation wavelength; and c is concentration of the fluorophore.

$$F_{adj} = F_{mea} \frac{\tau \cdot \lambda}{P^2 c} \quad (1)$$

Power was measured at the same sample plane using a calibrated power meter (Newport 818-ST). Values of τ were measured via intensity autocorrelation (Femtochrome FR-103TPM) every 50 nm from 750 nm to 1050 nm, and were interpolated to other wavelengths by natural cubic spline interpolation.

Two-photon excited fluorescence spectrum. The two-photon excited fluorescence spectrum was recorded using the same two-photon microscope setup except that the photomultiplier tube (PMT) was replaced by a fiber optic coupled spectrometer (Ocean Optics USB2000+). Figure S3 shows the two-photon excited fluorescence spectrum of BC575 in DMF, from excitation at 900 nm (excitation at other wavelengths from 800 - 1000 nm yielded nearly identical emission profiles).

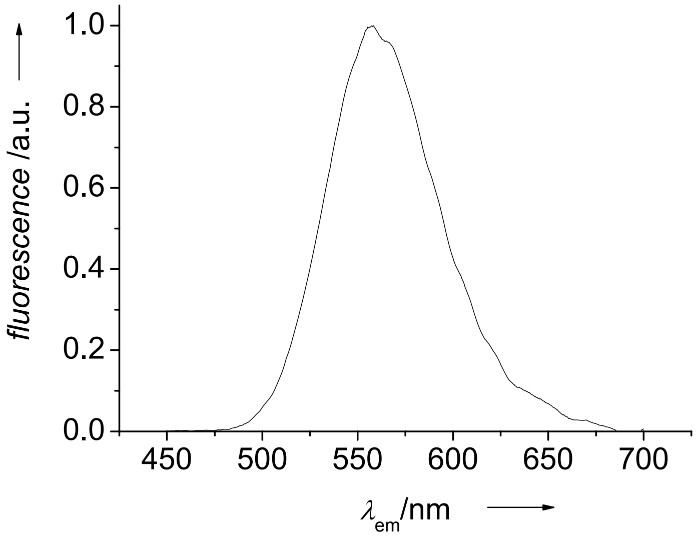

Figure S3. Two-Photon Excited Emission Spectrum. A solution of BC575 was excited by two-photon excitation. The graph shows the normalized fluorescence spectrum from excitation with a laser at 900 nm.

Fluorescent labeling

Cell Culture and Transfection of NIH3T3 Fibroblasts. NIH3T3 cells were cultured in Dulbecco's modified Eagle medium (DMEM) supplemented with newborn calf serum (NCS; 10%), L-glutamine (2 mM), penicillin and streptomycin, HEPES (15 mM), and incubated in a humidified atmosphere at 37 ºC and 5% CO_2. Adherent cells (ca. 80% confluent) were reseeded in a 8-well LabTek™ chambered coverglass (Nunc™); cells were transfected with plasmid DNA encoding NLS-eDHFR[1] using FuGENE HD

transfection reagent according to manufacture's instruction. Ca. 28 hr after transfection, cells were washed once with phosphate buffered saline (PBS) and then incubated with 1 μM TMP-BC575 in Ringer's buffer containing 10% NCS for 10 min at 37 ºC. Subsequently cells were washed 3 times and reimmersed in Ringer's buffer for confocal microscopy.

Cell Culture and Transfection of HEK293 Cells. HEK293 cells were cultured in high glucose DMEM supplemented with L-glutamine, pyruvate, and 10% fetal bovine serum (FBS), and incubated in a humidified atmosphere at 37 ºC and 5% CO_2. Adherent cells (ca. 80% confluent) were reseeded onto cover slides in 8-well plates; cells were transfected with 2 μg of plasmid DNA using FuGENE 6 (HEK293 cells) transfection reagent according to manufacture's instruction. Ca. 36 hr after transfection, cells were incubated in HEPES buffer and 1 μM TMP-BC575 for 10 min at 37 ºC. Then cells were washed once by HEPES buffer for two-photon microscopy.

Confocal microscopy

Confocal fluorescent microscopy of live cells was preformed using an Olympus IX81 scanning laser microscope equipped with a 60X Plan Fluor oil immersion objective. Laser excitation at 488 nm was used to excite the TMP-BC575 conjugate fluorescence. Differential interference contrast (DIC) and fluorescent images were captured by Fluoview FV500 software and processed using Image J. Figure S4 shows confocal fluorescence imaging of NIH3T3 cells expressing nucleus-targeted eDHFR and labeled by TMP-BC575.

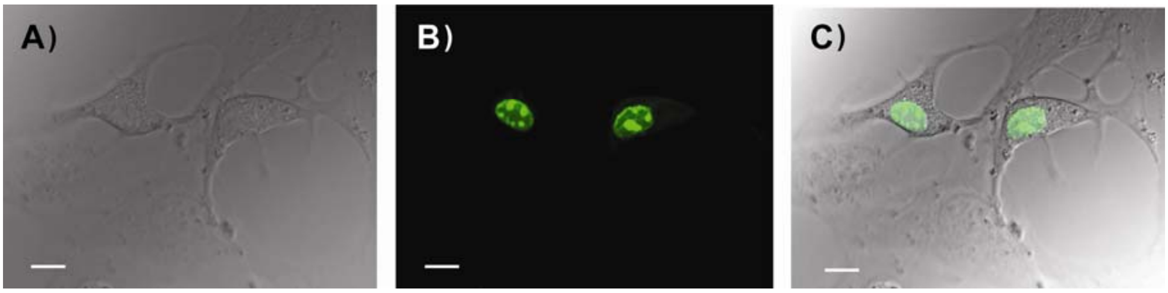

Figure S4. Selective Labeling of Intracellular Proteins by TMP-BC575. To evaluate the specificity of labeling, TMP-BC575 was used to label nucleus-targeted fusion proteins in wild-type fibroblasts. Cells transiently transfected with vector encoding the nucleus-targeted eDHFR fusion were incubated with 1 μM TMP-BC575 in Ringer's buffer containing 10% newborn calf serum for ten minutes, washed twice with phosphate buffered saline (PBS) and then imaged using live cell, confocal microscopy. A) - C) Confocal micrographs: A) differential image contrast; B) excitation of TMP-BC575 at 488 nm; C) overlay of A) and B). The labeling with TMP-BC575 showed distinct nuclear staining with no significant background fluorescence in the cytoplasm or untransfected cells. Scale bar: 10 μm.

Two-Photon Microscopy

The microscope setup for the two-photon fluorescence imaging has been previously described.[2] The microscope was also configured for oblique illumination with broadband infrared light, and can take transmission mode optical micrographs using a Hamamatsu EM-CCD (ImagEM C9100-13). Ultrafast laser excitation at 940 nm was used to excite the TMP-BC575 conjugate. Images were processed by ImageJ. Figure S5 shows oblique illumination (left) and two-photon imaging (right) of HEK293 cells transfected and untransfected with nucleus-targeted eDHFR and labeled by incubation with 1μM TMP-BC575. Figure S5C) shows the fluorescence signal intensities of images of transfected and untransfected cells.

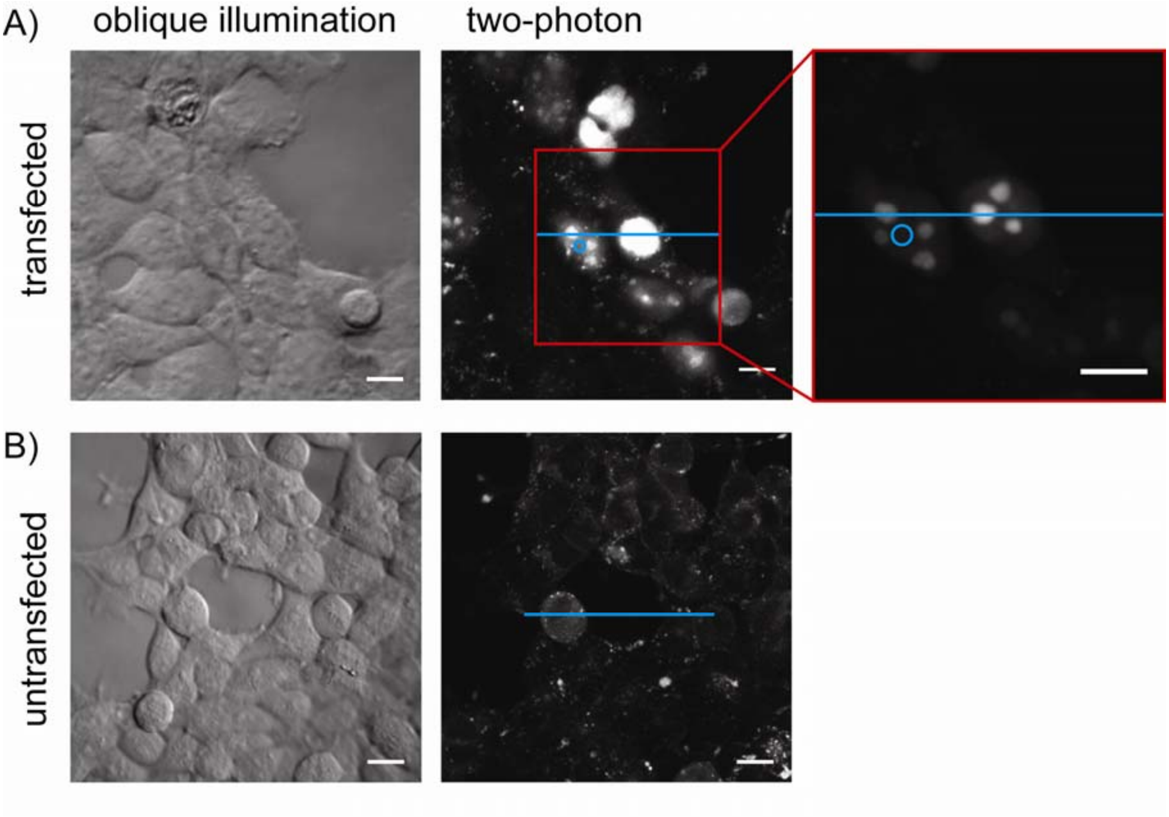

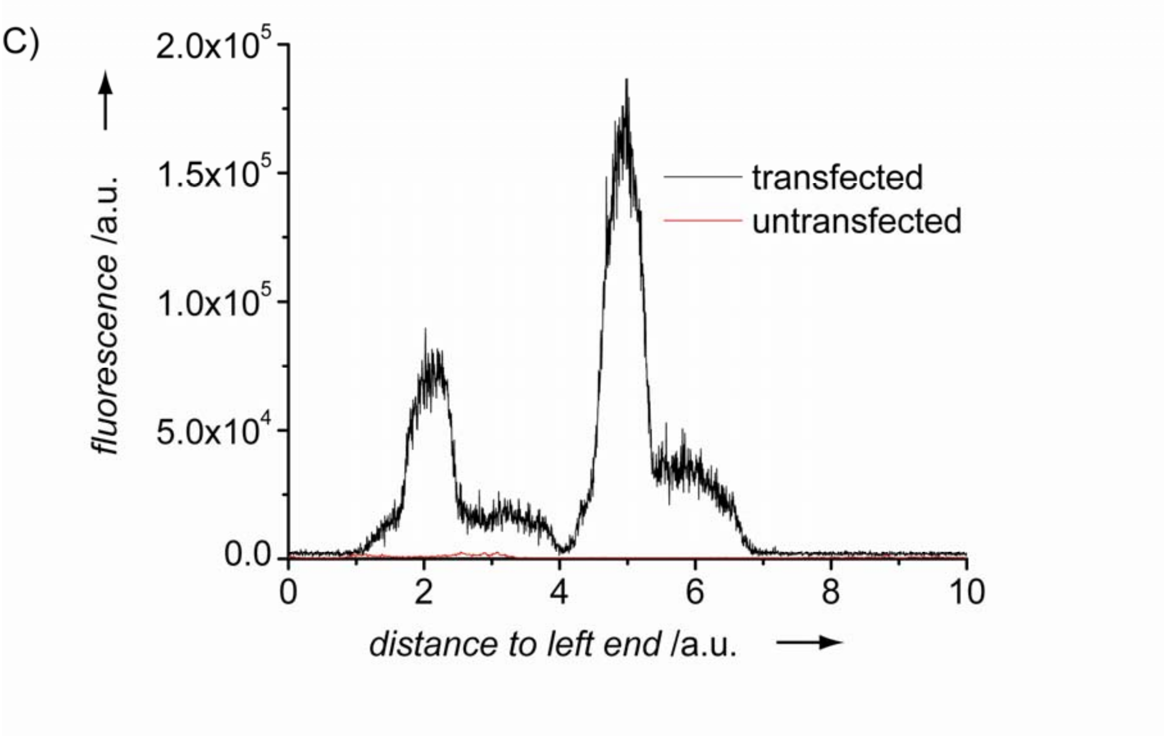

Figure S5. **Two-photon imaging of HEK293 cells**. Scale bar: 10 µm. A) - B) Oblique illumination (left column) and two-photon imaging (middle and right column) of HEK293 cells. Right column of A) is an image with higher resolution of highlighted region in middle column of A), using a lower excitation laser power and PMT voltage. A) HEK293 cells were transiently transfected with nucleus-targeted eDHFR and labeled with TMP-BC575. B) Untransfected HEK293 cells stained with TMP-BC575. C) Plot profile of two-photon micrographs of transfected (black line) and untransfected (red line) cells, showing the two-photon fluorescence signal intensity across the blue lines in A) and B), respectively. With laser powers sufficient to image the untransfected cells, the transfected cells have regions of severe saturation in the cell nucleus. To extend the effective dynamic range of our measurements, we acquired a transfected cell image at lower laser power (S5A, rightmost image) and scaled the intensity using the average intensity value of a shared region that was not saturated in either image shown by blue circles.

References

[1] N. T. Calloway, M. Choob, A. Sanz, M. P. Sheetz, L. W. Miller, V. W. Cornish. *Chembiochem* **2007**, *8*, 767-774.

[2] V. Nikolenko, B. Nemet, R. Yuste, *Methods* **2003**, *30*, 3-15.

[3] C. Xu, W. W. Webb, *J. Opt. Soc. Am. B* **1996**, *13*, 481-491 .
